# Secondary prevention of venous thromboembolism: Predictors and outcomes of guideline adherence in a long-term prospective cohort study

**DOI:** 10.3389/fcvm.2022.963528

**Published:** 2022-08-03

**Authors:** Tamara Mertins, Henning Nilius, Robin Boss, Matthias Knuchel, Andri Signorell, Carola A. Huber, Eva Blozik, Johanna Anna Kremer Hovinga, Lucas M. Bachmann, Michael Nagler

**Affiliations:** ^1^Department of Clinical Chemistry, Inselspital, Bern University Hospital, Bern, Switzerland; ^2^Department of Health Sciences, Helsana Group, Zurich, Switzerland; ^3^Institute of Primary Care, University of Zurich and University Hospital of Zurich, Zurich, Switzerland; ^4^Department of Hematology and Central Hematology Laboratory, Inselspital, Bern University Hospital, Bern, Switzerland; ^5^Faculty of Medicine, University of Bern, Bern, Switzerland; ^6^Medignition AG, Research Consultants, University of Zurich, Zurich, Switzerland

**Keywords:** venous thromboembolism, secondary prevention, health services research, guideline adherence, venous thrombosis - therapy

## Abstract

**Background:**

Prevention of recurrent venous thromboembolism (VTE) is considered a main goal of VTE management. However, the extent to which physicians adhere to the recommendations from evidence-based guidelines is unknown.

**Aim:**

From a large, prospective clinical cohort, we aimed to (1) quantify the adherence of treatment recommendations to evidence-based guidelines and establish its predictors, and (2) estimate its impact on clinical outcomes and costs in patients with VTE.

**Methods:**

We included 6'243 consecutive patients with VTE treated at the university outpatient unit. Detailed clinical characteristics and treatment recommendations were recorded. Adherence of treatment recommendations to evidence-based guidelines *at risk assessment* was assessed in terms of duration of anticoagulant treatment. Data on death were obtained from the Swiss Central Compensation Office. Health care claims data recorded between 2014 and 2019 were retrieved from Helsana, one of the largest Swiss health insurance companies.

**Results:**

The adherence to evidence-based guidelines was 36.1%. Among patients with non-adherence, overtreatment was present in 70.1%. Significant patient-related predictors of guideline adherence were (a) age above 50 years, (b) male sex, (c) pulmonary embolism, (d) unprovoked VTE, (e) multiple VTE, (f) laboratory tests not ordered, and (g) various cardiovascular comorbidities. Non-adherence was not significantly associated with mortality, hospitalization, admission to nursing home, and costs.

**Conclusions:**

The adherence to evidence-based guidelines was low, and several unrelated predictors appeared. Although these results need to be confirmed in other settings, they highlight the need for implementation of evidence-based guidelines in clinical practice.

## Introduction

Venous thromboembolism (VTE) contributes significantly to the global disease burden (1). The incidence is estimated to be 1.5 million per year within the European Union (2). In the United States, the annual healthcare costs are estimated to be seven to nine billion US$ (3). Secondary prevention of VTE is considered the most important measure to reduce its impact on society (4). Risk assessment for recurrent VTE is recommended by evidence-based guidelines and long-term anticoagulation treatment is recommended in patients at high risk (4, 5). Direct oral anticoagulants have made treatment safer and considerable simpler (6–11). However, the extent to which this evidence translates into clinical practice is largely unclear (9, 12–14). Inadequate application of evidence-based guidelines might substantially reduce the quality of care in patients with VTE (12, 15–18). Studies investigating the clinical practice of secondary prevention of VTE are scarce, and the degree of guideline adherence is essentially unclear (13).

In a single-center prospective cohort study, we aimed to (1) observe the adherence of treatment recommendations to evidence-based guidelines and establish its predictors, and (2) estimate its impact on clinical outcomes and costs in patients with VTE.

## Materials and methods

### Study design, setting, and population

**Figure 1:**
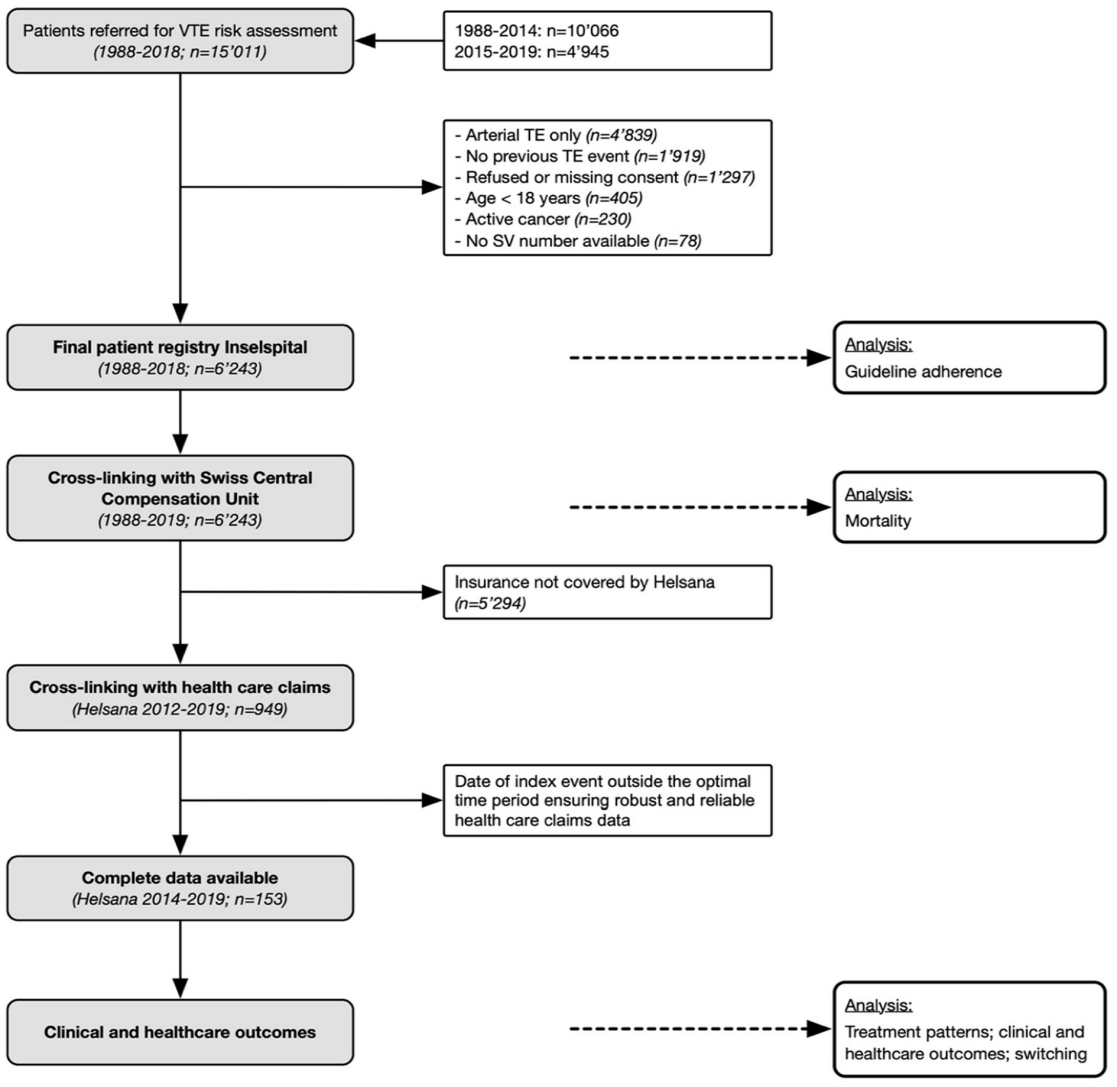
Flow of the patients. In a long-term prospective cohort study including patients with VTE, the adherence of treatment recommendations with evidence-based guidelines were studied, predictors established, treatment patterns observed, and various outcomes studied.

This analysis is part of a long-term, prospective cohort study (“SeProV”), details of which are described previously (19). Consecutive patients, referred between 1988 and 2018 for VTE risk assessment to a specialized outpatient unit at the Inselspital, Bern University Hospital, were included. Inclusion criteria were (1) objectively confirmed VTE, (2) referral for VTE risk assessment, and (3) age above 18 years. Exclusion criteria were (a) refused informed consent (b) active cancer, and (c) arterial thromboembolism only (Figure 1). The Inselspital Bern is one of the largest university hospitals in Switzerland. It has a catchment area of about 2 million inhabitants, including both German-speaking and French-speaking areas. In all patients considered for secondary prevention of VTE, a formal risk assessment is recommended, and the specialized outpatient unit is the most important center in the greater Bern area. Therefore, we assume that most patients with VTE were referred to our center. All patients consented to data collection either by informed consent (since 2003) or by non-refusal (1988 to 2003). The study was conducted in accordance with the Declaration of Helsinki. The Local Ethical Committee approved the study protocol and data collection (Kantonale Ethikkommission Bern; No. 18-00389).

### Work-up of patients and data collection

Patients were referred from general practitioners, hospital specialists, or vascular specialists. Medical records were requested beforehand. A detailed medical history, including family history, was obtained by a specialist registrar using a structured assessment form. A focused clinical examination was performed. The following pre-defined data were collected: age, sex, type of index VTE, triggering clinical risk factors, arterial thrombosis. As additional risk factors, the following variables were collected: smoking status, obesity, presence of systemic disease, inflammatory bowel disease, systemic lupus erythematosus, diabetes, coronary artery disease without myocardial infarction, chronic lung disease, hypertension, active cancer, kidney failure, and anemia. Laboratory tests including thrombophilia markers were ordered at the discretion of the attending physician. Using this structured data and laboratory test results, the individual risk for recurrent VTE was interpreted. This interpretation was challenged by the senior physician, and possibly discussed with the full team at a designated board meeting. By means of this process, the intended duration of anticoagulation was defined and communicated to the family physician. Except for adverse events, family physicians adhere to this recommendation. Data were extracted from the assessment forms routinely into a designated database. These data were additionally checked by a trained investigator and a specialized study nurse. Requested laboratory test were grouped into the following categories: (a) thrombophilia markers (activated protein C resistance, factor V-Leiden mutation, prothrombin gene mutation, protein S deficiency, protein C deficiency, antithrombin deficiency), (b) coagulation tests (prothrombin time, activated partial thromboplastin time, fibrinogen concentration, thrombin time, factor II, factor V, factor VII, factor X), (c) antiphospholipid tests (lupus anticoagulant, anticardiolipin antibodies, beta-2-glycoprotein antibodies), (d) D-dimers and factor VIII, and (e) full blood count.

### Cross-linking with swiss central compensation unit

The database was matched with the Unique Person Identification registry of the Swiss Central Compensation Unit (ZAS) to obtain the vital status and the time point of death (Figure 1) (19). The database is regarded as complete because all Swiss civil registries report to the ZAS. The Swiss social insurance number (SV) was used for matching. Patients moved abroad (not covered by ZAS) were censored.

### Health care claims data

The database was matched with Helsana health care claims received between 1st of January 2014 and 31st June 2019 (Figure 1). Data before 2014 were not eligible due to changes in the Swiss inpatient reimbursement system and thus excluded in our analysis. Helsana group is one of the largest health insurance companies in Switzerland. Since the content of the statutory health insurance is determined by law in Switzerland, there exist no differences between Helsana and other Swiss health insurances. Patients insured by Helsana can be deemed representative of the Swiss population. A specialized department records health care claims in a structured, high-quality database. These data have already been used in a large number of studies (e.g. 58, 59). The matching of data was based on the SV number. It conforms with privacy protection regulations and Swiss law on human research, and it was approved by the appropriate Ethical Committee.

### Definition of variables

The type of the index event and the triggering risk factors were defined following current guidelines (19, 20). The appropriateness of this categorization was verified again in 2018 using the original patient records. Mutually exclusive groups were created regarding the type of the index event, considering the most severe thromboembolic event only (pulmonary embolism [PE] in case of multiple VTE). Patients with any type of deep vein thrombosis (DVT; lower leg, proximal, pelvic) without PE were categorized as DVT. All other VTE were classified as “other VTE” (cerebral vein thrombosis, mesenteric thrombosis, portal vein thrombosis, upper extremity deep vein thrombosis, superficial vein thrombosis, muscle vein thrombosis).

Unprovoked VTE was defined as the absence of reversible risk factors (surgery, traumatic injury at most 3 months before the VTE, catheter, immobilization, contraceptive use, pregnancy, long-distance travel of more than 10 h) (20, 21). Surgery, immobilization, long-distance travel of more than 10 h and traumatic injury within 3 months before the VTE were classified as transient risk factors. Pregnancy and anti-contraceptive use (estrogen-containing) were classified as hormone-related risk factors. Duration of anticoagulant treatment was categorized as follows: (1) < 6 months, (2) seven Helsana group 24 months (prolonged), >24 months (permanent) (19). Patients were categorized as obese if BMI >30 kg/m^2^. All other comorbidities were recorded as either present or absent: arterial thrombosis, coronary artery disease, hypertension, pulmonary disease, diabetes mellitus, stroke, coronary heart disease, kidney disease, and anemia. Patients' smoking status was recorded as either active smokers or non-smokers.

### Assessment of guideline adherence

The adherence of treatment recommendations to evidence-based guidelines at risk assessment was assessed in terms of duration of anticoagulant treatment, according to type of event and risk factor. We determined whether the treatment recommendation was done in accordance with the current guidelines in pre-defined risk groups (unprovoked proximal DVT & PE; multiple VTE, provoked DVT, distal DVT, and VTE during pregnancy). Other patients were not considered. Since no specific Swiss guideline is available, we used the following international guidelines: The American College of Chest Physicians [ACCP; 2001 (22), 2004 (23), 2008 (24), 2012 (25), 2016 (26)], the British Committee for the Standards in Hematology [BCSH; 1998 (27), 2001 (28)], and Deutsche Gesellschaft für Angiologie [DGA; 2010 (29); 2015 (30)]. The treatment recommendation for each risk category was recorded per period (Table 1). Then, we assessed whether the treatment recommendation followed the respective guideline for each patient. The overall adherence was determined for all guidelines, and the guideline with the highest agreement was used for all other analyses. Adherence was as assessed in salient subgroups of patients (defined above). Overtreatment was defined as a longer treatment period than recommended, and undertreatment as a shorter treatment period.

**Table 1:** Treatment recommendations in the secondary prevention of venous thromboembolisms.

**Guideline**	**Year**	**Management recommendations**				
		**Unprovoked** **proximal DVT & PE**	**Multiple VTE**	**Provoked proximal** **DVT & PE**	**Distal DVT**	**VTE during pregnancy**
ACCP	2001	6–12 months	Extended	3–6 months	3 months	3 months
	2004	Extended	Extended	3 months	Same as proximal DVT	3 months
	2008	Extended	Extended	3 months	3 months	3 months
	2012	Extended	Extended	3 months	3 months	3 months
	2016	Extended	Extended	3months	3 months	3 months
BCSH	1998	Extended	–	6 months	3 months	–
	2001	6 months	Extended	3 months	<6 months	6 months
DGA	2010	>3 months	Extended	3 months	3 months	6 weeks postpartum
	2015	>6 months	>6 months	3 months	3 months	6 weeks postpartum

Guidelines used in Switzerland since 2001 are shown. The duration of anticoagulation treatment in major risk categories is given. The American College of Chest Physicians [ACCP; 2001 (22), 2004 (23), 2008 (24), 2012 (25), 2016 (26)], the British Committee for the Standards in Haematology [BCSH; 1998 (27), 2001 (28)], and Deutsche Gesellschaft für Angiologie [DGA; 2010 (29); 2015 (30)].

### Statistical analysis

Patient characteristics were calculated by sex and presented as either number (%) or median (interquartile range) as appropriate. A small number of missing values, considered to be missing at random, were imputed using a random-forest-based algorithm [Table 2; “Missforest” package for “R” (31)]. The algorithm can handle categorical and numerical values and works similarly to other modern imputation methods. Patients which were not covered by the respective guideline were excluded from analysis. Multiple logistic regression models were created, using the parameters as independent variables and guideline adherence as the dependent variable. The regression coefficients were reported as odds ratios (OR) and corresponding 95% confidence intervals (CI). Multivariate logistic regression models were adjusted for risk factors, type of thrombosis, and multiple VTE. Multivariate cox-proportional hazard regression models were done for mortality, hospitalization, and nursing home entry. Guideline adherence was entered as the independent variable, death, hospitalization, or nursing home entry as the dependent variable. The models were adjusted for age, sex, cardiovascular comorbidities, type of thrombosis, multiple VTE, and potential risk factors. The influence of guideline adherence on the health care costs was analyzed through a linear model, that was also adjusted for age, sex, cardiovascular comorbidities, type of thrombosis, multiple VTE, and risk factors. All analyses were done using the “stats,” and “survival” for “R” version 4.0.5. (32–34).

**Table 2:** Patient characteristics.

	**Female**	**Males**	**Overall**	**Missing values (%)**
n	3,649	2,594	6,243	
Median age *(IQR)*	41.64 (29.4, 52.8)	49.37 (40.11, 59.19)	44.86 (32.6, 56.1)	
**Type of thrombosis** *(numbers, %)*				30 (0.4)
Proximal DVT	668 (18.4)	455 (17.6)	1,123 (18.1)	
Distal DVT	855 (23.6)	528 (20.4)	1,383 (22.3)	
Pulmonary embolism	1,431 (39.4)	1,248 (48.3)	2,679 (43.1)	
Other	676 (18.6)	352 (13.6)	1,028 (16.5)	
**OAK duration**				7 (0.1)
<3 months	562 (15.4)	239 (9.2)	801 (12.8)	
3–6 months	1,790 (49.1)	1,026 (39.6)	2,816 (45.2)	
> 6 months	518 (14.2)	315 (12.2)	833 (13.4)	
Extended	777 (21.3)	1009 (39.0)	1,786 (28.6)	
**Triggering risk factor**				30 (0.4)
Provoked	2,322 (63.9)	945 (36.6)	3,267 (52.6)	
Unprovoked	992 (27.3)	1,630 (63.2)	2,622 (42.2)	
Pregnancy	324 (8.8)	0 (0.0)	324 (5.2)	
Multiple VTE	949 (26.1)	997 (38.5)	1,946 (31.2)	15 (0.2)
Family history	1,176 (32.4)	792 (30.6)	1,968 (31.6)	24 (0.4)
Arterial thromboembolism	124 (3.4)	161 (6.2)	285 (4.6)	0 (0)
Smoking	839 (23.3)	606 (23.7)	1,445 (23.5)	87 (1.4)
Obesity	992 (27.7)	800 (31.2)	1,792 (29.2)	99 (1.6)
System diseases	216 (5.9)	160 (6.2)	376 (6.0)	16 (0.3)
Diabetes	79 (2.2)	92 (3.6)	171 (2.7)	18 (0.3)
CAD	89 (2.4)	170 (6.6)	259 (4.2)	18 (0.3)
Lung disease	140 (3.8)	111(4.3)	251 (4.0)	17 (0.3)
Hypertonus	372 (10.2)	431 (16.7)	803 (12.9)	18 (0.3)
Kidney failure	56 (1.5)	83 (3.2)	139 (2.2)	16 (0.3)
Stroke	89 (2.4)	102 (3.9)	191 (3.1)	17 (0.3)
Anemia	160 (4.4)	57 (2.2)	217 (3.5)	17 (0.3)
**Laboratory tests ordered**				
Blood count	754 (20.7)	405 (15.6)	1,159 (18.6)	0 (0)
Coagulation tests	3,191 (87.4)	2,174 (83.8)	5,365 (85.9)	0 (0)
D-dimers	3,029 (83.0)	2,230 (86.0)	5,259 (84.2)	0 (0)
Antiphospholipid tests	2,447 (67.1)	1,723 (66.4)	4,170 (66.8)	0 (0)
Thrombophilia markers	3,294 (90.3)	2,284 (88.0)	5,578 (89.3)	0 (0)

## Results

### Characteristics of patients

Out of 15'011 patients screened between 1988 and 2018, we finally included 6'243 consecutive patients with venous thromboembolism (Figure 1). Detailed patient characteristics are shown in Table 2. The median age was 44.86 years (interquartile range [IQR] 32.6, 56.1), 3'649 patients were female (58.4%). Female patients were younger (median 41.6 years, IQR 29.4, 52.8) than male patients (median 49.4, IQR 40.1, 59.2).

The type of venous thromboembolic events was pulmonary embolism in 2'679 patients (43.1%), proximal DVT in 1'123 individuals (18.1%), distal DVT in 1'383 cases (22.3%), and other VTE in 1'028 patients (16.5%). Unprovoked VTE was identified in 2'622 (41.9%), and provoked VTE was observed in 3'267 patients (52.6%). Pregnancy-related VTE was present in 324 patients (5.2%). The duration of oral anticoagulation (OAK) was <3 months in 801 cases (12.8%), 3–6 months in 2'816 cases (45.2%), >6 months in 833 cases (13.4%) and extended in 1'786 cases (28.6%). Multiple previous VTE were present in 1'946 patients (31.2%), and a positive family history of VTE was observed in 1'968 individuals (31.6%).

Thrombophilia markers were requested in 5'578 patients (89.3%), coagulation tests in 5'365 individuals (85.9%), antiphospholipid tests in 4'170 patients (66.8%), blood count in 1'159 cases (18.6%), and D-dimers in 5'259 patients (66.8%).

### Adherence to evidence-based guidelines

The adherence of treatment recommendations to evidence-based guidelines was 32.2% in the case of the ACCP, 33.0% in BCSH, and 36.1% in DGA (Supplementary Figure S1). Non-adherence was present in 56.2 % in case of ACCP, 44.1% in BCSH, and 52.2 % in DGA. Patients that were not covered by the guidelines were 11.7% in case of the ACCP, 23.0% in case of the BCSH and 11.7% in case of the DGA. Following these results, we applied the DGA guideline for all subsequent analyses. In patients with non-adherence, overtreatment was observed in 70.1 %, and undertreatment in 29.9 %.

### Predictors of guideline adherence

**Figure 2:**
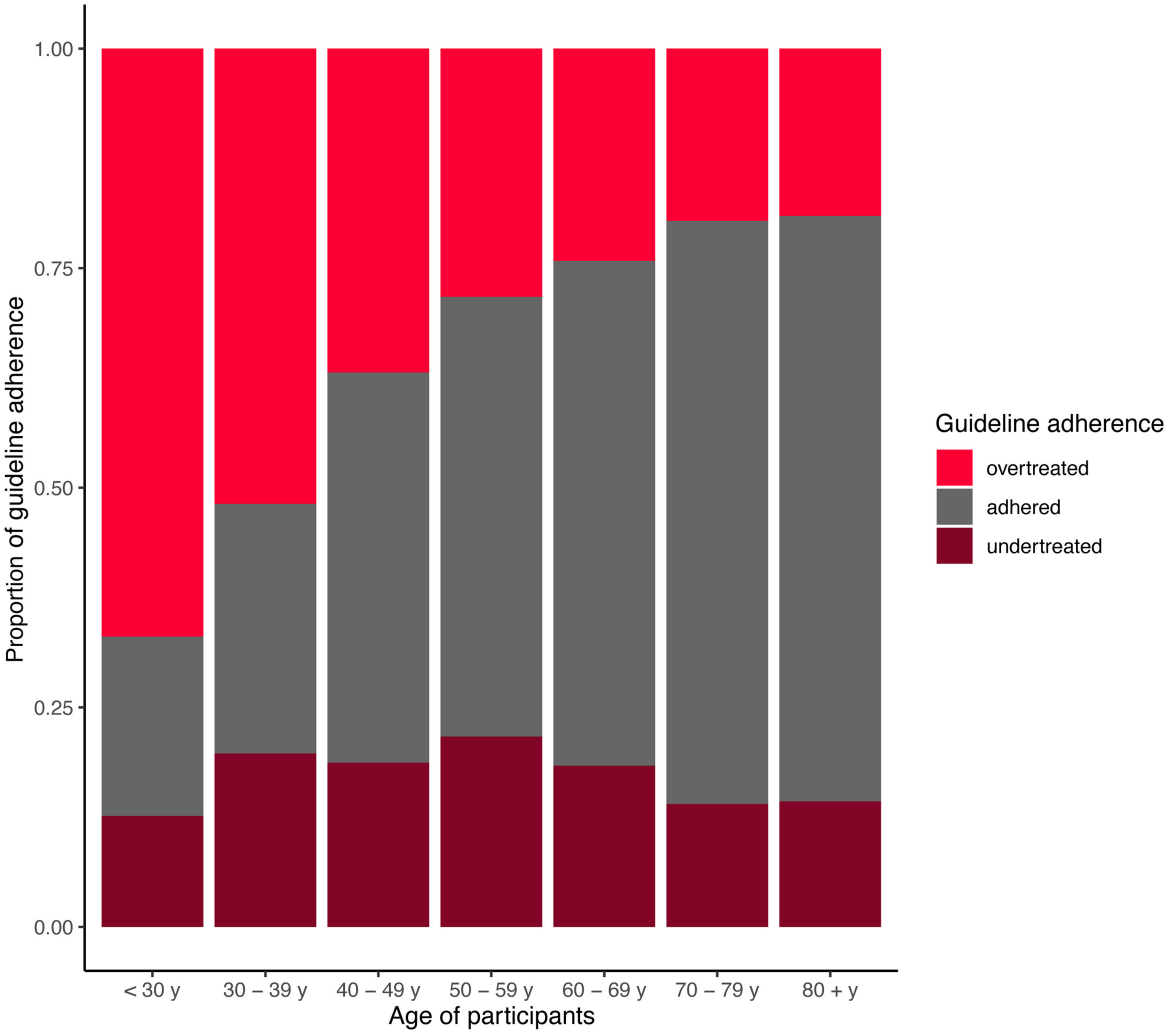
Adherence to evidence-based guidelines according to patients' age. Proportions are given.

The guideline adherence according to age is illustrated in Figure 2. The percentage of guideline adherence increased steadily from 20.9% in patients <30 years of age to 66.7 % in patients >80 years of age. The percentage of patients overtreated was highest in patients <30 years of age (67.1%), and lowest in patients >80 years of age (23.8%). In contrast, the proportion of patients undertreated was stable and ranged between 12.0 % (<30 years) and 21.6 % (50–59 years).

A broad range of potential predictors of guideline adherence is given in Table 3. The following factors were significantly associated with adherence: age above 50 years (OR 2.56; 95% CI 2.29, 2.87), male sex (OR 2.66; 95% CI 2.38, 2.97), pulmonary embolism (OR 1.40, 95% CI 1.22, 1.61), unprovoked VTE (OR 10.77, 95% CI 9.47, 12.28), multiple VTE (OR 2.36; 95% CI 2.11, 2.64), coronary artery disease (OR 2.50; 95% CI 1.92, 3.27), kidney failure (OR 2.37; 95% CI 1.65, 3.43), and various cardiovascular risk factors.

**Table 3:** Predictors of guideline adherence in a single center prospective cohort study (*n* = 6'243).

	**OR**	**95% CI**	**Adjusted OR**	**Adjusted 95% CI**
Age^*^				
<50 y	ref	ref	ref	ref
≥50 y	2.56	2.29, 2.87	1.43	1.24, 1.64
Sex^*^				
Female	ref	ref	ref	Ref
Male	2.66	2.38, 2.97	1.26	1.09, 1.45
Type of thrombosis^*^				
Proximal DVT	ref	ref	ref	ref
Distal DVT	0.51	0.43, 0.61	0.41	0.34, 0.52
Pulmonary embolism	1.40	1.22, 1.61	1.37	1.13, 1.59
Other	0.70	0.53, 0.91	0.21	0.16, 0.30
Triggering risk factors^*^				
Provoked	ref	ref	ref	ref
Unprovoked	10.77	9.47, 12.28	11.12	9.69, 12.78
Pregnancy	1.38	1.05, 1.80	1.66	1.24, 2.20
**Other factors**				
Multiple VTE	2.36	2.11, 2.64	2.33	2.02, 2.69
Family history	1.01	0.90, 1.13	0.98	0.86, 1.14
Arterial thromboembolism	1.64	1.27, 2.13	1.43	1.05, 1.96
Smoking	0.88	0.78, 1.00	0.97	0.83, 1.14
Obesity	1.34	1.19, 1.51	1.12	0.96, 1.29
System diseases	1.69	1.36, 2.12	1.48	1.13, 1.95
Diabetes	1.70	1.23, 2.36	1.17	0.79, 1.74
CAD	2.50	1.92, 3.27	1.75	1.26, 2.43
Lung disease	1.22	0.94, 1.60	0.98	0.71, 1.35
Hypertonus	1.71	1.46, 2.00	1.20	1.00, 1.45
Kidney failure	2.37	1.65, 3.43	1.53	1.00, 2.35
Stroke	1.44	1.06, 1.96	1.37	0.95, 1.96
Anemia	0.92	0.68, 1.24	0.76	0.52, 1.09
**Laboratory tests ordered**				
Blood count	0.61	0.53, 0.70	0.56	0.47, 0.66
Coagulation tests	0.69	0.59, 0.80	0.89	0.74, 1.08
D-dimers	1.24	1.07, 1.44	1.33	1.11, 1.59
Antiphospholipid tests	0.77	0.69, 0.86	0.74	0.64, 0.85
Thrombophilia markers	0.69	0.58, 0.82	0.86	0.70, 1.06

Unadjusted and adjusted odds ratios are given in comparison to a reference group or absence of the factor.

* Mutual exclusive groups.

**Figure 3:**
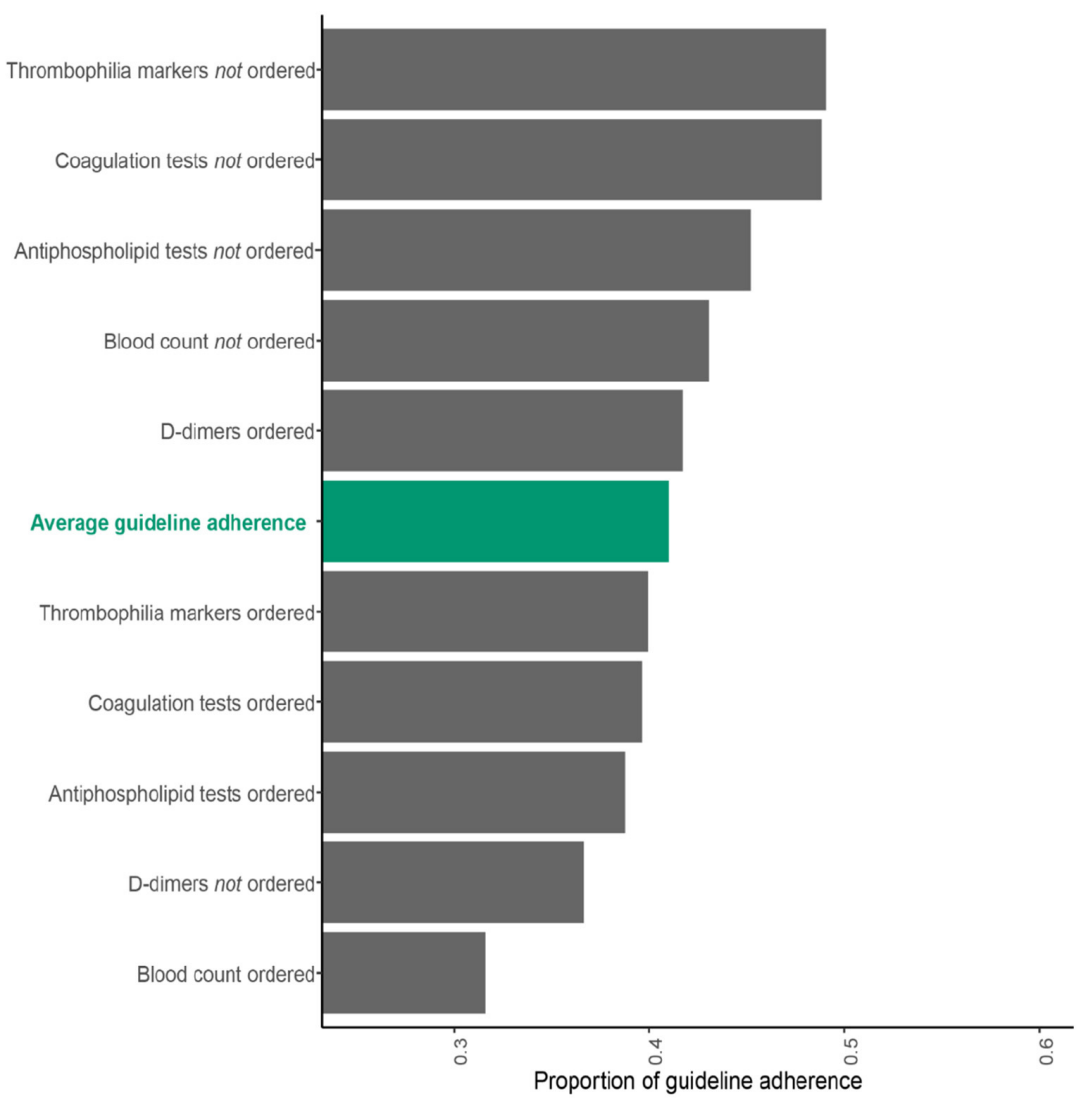
Guideline adherence in the secondary prevention of VTE depending on whether laboratory tests were ordered. Data of a single-center prospective cohort study are shown (*n* = 6'243). Proportions are given, the average guideline adherence is illustrated in green.

An association between lab test ordering and guideline adherence was observed, details are given in Figure 3. Whereas the ordering of D-dimers was associated with a higher guideline adherence (OR 1.24; 95% CI 1.07, 1.44), were all other laboratory tests associated with a lower guideline adherence: (blood count (OR = 0.61; 95% CI 0.53, 0.70), coagulation status (OR = 0.69; 95% CI 0.59, 0.80), antiphospholipid workup (OR 0.77; 95% CI 0.69, 0.86), and thrombophilia markers (OR = 0.69; 95% CI 0.58, 0.82).

### Treatment patterns

Of the 6'243 patients, 949 had health insurance with Helsana and matching could be performed (Figure 1). However, the index event was in the optimal time period (2014–2019) in 153 patients only. The pattern of treatment was consistent with the initial treatment recommendation in 126 of 153 cases (82.4%). Permanent anticoagulation treatment was done in 61 patients (39.9%). DOAC were used in 42 cases (68.9%), and VKA in 21 (13.7%). Switching from VKA to DOAC was observed in two patients (3.3%), and from VKA to DOAC in one patient (1.6%).

### Clinical and healthcare outcomes

Two-hundred and forty-three deaths occurred among 6'243 patients cross-linked with the ZAS (Figure 1). The mortality rate was 0.62 per 100 patient-years in patients adhered to evidence-based guidelines and 0.35 in patients not adhered (Table 4). The hazard ratio of a cox proportional hazards model adjusting for age, sex, cardiovascular comorbidities, type of thrombosis, multiple VTE, and triggering risk factors was 0.89 (95% CI 0.83, 1.52). Health care claims data were used to assess the associations between guideline adherence and hospitalizations, admission to nursing home, and costs (Table 4; *n* = 153). Considering subsequent hospitalizations, the HR was 1.04 (95% CI 0.49, 1.88; Figure 1). We were unable to calculate the hazard for admission to nursing home because of the low number of events (3 vs. 1). The median costs were higher in patients adhered to guidelines, but the confidence intervals were wide.

**Table 4:** Association of guideline adherence with clinical and healthcare outcomes in patients with VTE.

**Outcome**	**Number of patients**	**Adhered to** **guidelines**	**Not adhered**	**Association** **adhered vs. not adhered**
		**Events per 100** **patient-years**	**Events per 100** **patient-years**	**(95% confidence interval)**
Mortality	6'243^+^	0.62	0.35	HR 0.89 (0.83, 1.52)^*^
Hospitalization	153^+^	34.0	30.8	HR 1.04 (0.49, 1.88)^*^
Admission to nursing home	153^+^	2.32	0.71	N/A^#^
Costs (CHF; median, IQR)	153^+^	24'644 (14'151, 77'116)	21'403 (9'261, 38'658)	1'689^$^ (−35'160.16, 38'538.61)

Data of a long-term prospective cohort study are shown.

* HR, hazard ratio of a cox proportional hazards model adjusting for age, sex, cardiovascular comorbidities, type of thrombosis, multiple VTE and triggering risk factors; patients adhered vs. not adhered;

+ patients not covered by current guidelines were not considered for analysis;

# Cannot be calculated due to the limited number of events (3 vs. 1);

$ Mean difference adhered vs. not adhered in a linear regression model adjusting for age, sex, cardiovascular comorbidities, type of thrombosis, multiple VTE and triggering risk factors.

## Discussion

In a single-center prospective cohort study, we assessed the adherence to evidence-based guidelines at risk assessment. Overall, 6'243 patients were included between 1988 and 2018, and detailed clinical data were obtained. The patients were matched with the Swiss Central Compensation Unit and health care claims data. The adherence of treatment recommendations to evidence-based guidelines was 36.1%. Non-adherence was caused by overtreatment in 70.1%, and undertreatment in 29.9%. Significant predictors of guideline adherence were (a) age above 50 years, (b) male sex, (c) pulmonary embolism, (d) unprovoked VTE, (e) multiple VTE, (f) laboratory tests not ordered, and (g) various cardiovascular comorbidities. Non-adherence was not significantly associated with mortality, hospitalization, admission to nursing home, and costs.

We are not aware of previous studies analyzing the adherence to evidence-based guidelines in the secondary prevention of VTE. However, several studies assessed other aspects of VTE prevention and management, and the results are in line with our study. Three extensive cross-sectional surveys found a low rate of appropriate VTE prophylaxis in hospitalized patients (35–37). Kucher and colleagues identified predictors of appropriate VTE prophylaxis in hospitalized individuals (16). In an analysis of the RIETE registry, Roldan et al. found that a substantial proportion of thrombophilia orderings were inconsistent with current guidelines (14). A survey among Dutch practitioners found a wide variety of considerations regarding treatment duration in patients with unprovoked VTE (18). In contrast to these data, a high proportion of patients received adequate long-term anticoagulation in 52 German family practices (17). Recently, the appropriateness of thrombophilia testing was observed in the inpatient setting (38). Significant over- and undertreatment was also observed in a survey among hematologists and respiratory physicians in Australia (39). In another analysis of the RIETE registry, patients with non-recommended dosing were at 10 times higher risk of recurrent VTE (40).

The strength of our investigation is that we included a large number of patients, which were observed over a long period of time. Therefore, not only can adherence be studied very precisely, but also a whole set of predictors can be established. Another strength is that we were able to link data from 6,243 patients to the ZAS, allowing us to study mortality in detail. Another strength is that we have paid a significant amount of attention to accurate and comprehensive measurements to avoid residual confounding. However, to achieve this, we had to limit the period for retrieving health care claims data to a robust and reliable range between 2014 and 2018, covering only 153 patients (949 out of 6'243 were insured with Helsana). As a result, there is very little information on patients who have discontinued treatment or changed the type of treatment. As another important limitation, the data were collected in one institution only, and it is likely that the extent of adherence varies between different institutions. However, the results are consistent with studies that examined adherence to guidelines in other aspects of VTE management. As a third limitation, the patient inclusion was ended in 2018, and we cannot fully exclude that the adherence would be better recently. Fourthly, the median age of the patients in our cohort was rather low (44.9 years), which hints to a certain degree of selection bias in terms of younger patients. Thus, the degree of adherence might be better in an older population. Fifthly, the individual bleeding risk and the personal preferences were not recorded in a structured way. But even assuming that a relevant proportion of patients will have such problems and the results will be biased to a certain degree, the general interpretation will still be the same: that guideline adherence is low in clinical practice. Further, one could be argued that these patients with non-adherence represent exceptional situations with high-risk thrombophilia, for which the guidelines allow room for interpretation. However, the incidence of these conditions is very low based on the current literature and cannot explain the large proportion of non-adherent patients in our study (41). One observation should be commented, which seems unusual at first sight: the mortality rate was lower in patients who adhered to the guidelines. We believe that this is a case of unmeasured confounder. Patients with more concomitant diseases (or sicker patients) were more likely to be treated according to guidelines than patients without. The predictors in Table 3 and the costs also point in this direction. Despite multivariate analysis, we were not able to fully avoid this effect.

In the absence of other studies, our results suggest that guideline adherence is low in the secondary prevention of VTE. Physicians often do not follow guidelines when making clinical decisions (42). However, future studies shall confirm our results in other settings and institutions. Our results call for efforts to improve guideline adherence in the management of VTE. This might relevantly improve care in patients with VTE.

## Conclusions

Without more extensive and generalizable observational studies, our study suggests low adherence to evidence-based guidelines in patients with VTE. Significant predictors of guideline adherence were (a) age above 50 years, (b) male sex, (c) pulmonary embolism, (d) unprovoked VTE, (e) multiple VTE, (f) laboratory tests not ordered, and (g) various cardiovascular comorbidities. Our results call for efforts to improve guideline adherence in the secondary management of VTE. This might relevantly improve care in many patients.

## Data availability statement

The raw data supporting the conclusions of this article will be made available by the authors, without undue reservation.

## Ethics statement

The studies involving human participants were reviewed and approved by Kantonale Ethikkommission Bern. The patients/participants provided their written informed consent to participate in this study.

## Author contributions

TM collected data, contributed to the analysis and interpretation, and wrote the manuscript. HN conducted the analysis and interpreted the data. RB and MK collected data and contributed to interpretation and manuscript. AS contributed to study design, collected data, and analyzed the data. CH, EB, JK, and LB contributed to study design and manuscript. MN designed the study, analyzed the data, interpreted the findings, and wrote the manuscript. All authors contributed to the article and approved the submitted version.

## Funding

This investigator-initiated study was supported by an unrestricted research grant of Bayer Healthcare. MN was supported by a research grant of the Swiss National Science Foundation (#179334).

## Conflict of interest

This study received funding from an unrestricted research grant of Bayer Healthcare. The funder was not involved in the study design, collection, analysis, interpretation of data, the writing of this article or the decision to submit it for publication. EB reports grants from Novartis, grants from MSD, grants from Vifor, grants from Bayer, grants from Swiss Cancer Research Foundation, outside the submitted work. JK reports grants from Baxalta US Inc., member of the Takeda group of companies, personal fees from Shire, member of the Takeda group of companies, personal fees from Ablynx, now part of Sanofi, personal fees from Roche, from SOBI, from Federal Office of Public Health, Switzerland, outside the submitted work; and The Hemophilia Comprehensive Care Center (HCCC) is part of the Department of Hematology and Central Hematology Laboratory, Inselspital, Bern University Hospital, which receives third party funds for the project “Interprofessional Hemophilia Care” by Bayer, CSL-Behring, Octapharma, Novo Nordisk, Roche, and Sobi. All fees or honoraria go to JK's institution Insel Gruppe AG, Inselspital, Bern University Hospital. The remaining authors declare that the research was conducted in the absence of any commercial or financial relationships that could be construed as a potential conflict of interest.

## Publisher's note

All claims expressed in this article are solely those of the authors and do not necessarily represent those of their affiliated organizations, or those of the publisher, the editors and the reviewers. Any product that may be evaluated in this article, or claim that may be made by its manufacturer, is not guaranteed or endorsed by the publisher.

## Supplementary material

Supplementary Figure S1 Adherence to different guidelines used in the secondary prevention of VTE in terms of treatment recommendations. Data of a single-center prospective cohort study in Switzerland are shown (*n* = 6'243). Proportions are given (red, not adhered; dark grey, adhered; light grey, not covered).
