# Supplementary figures and images for: Secondary prevention of venous thromboembolism: Predictors and outcomes of guideline adherence in a long-term prospective cohort study
Source: Front Cardiovasc Med. 2022 Aug 3;9:963528. doi: 10.3389/fcvm.2022.963528 (PMC9381867; doi:10.3389/fcvm.2022.963528)

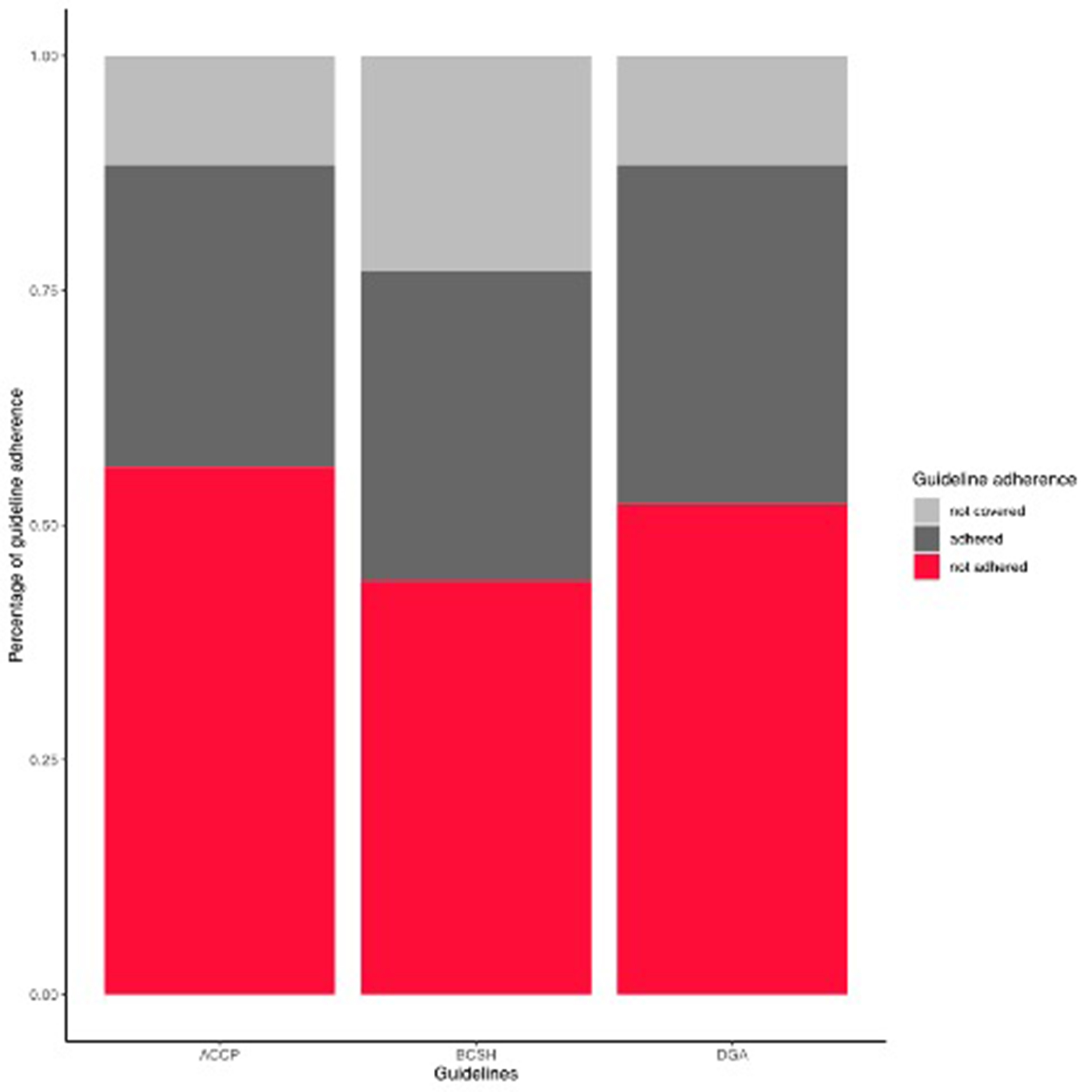

Supplement: Supplementary Figure S1 — Adherence to different guidelines used in the secondary prevention of VTE in terms of treatment recommendations. Data of a single-center prospective cohort study in Switzerland are shown (n = 6'243). Proportions are given (red, not adhered; dark grey, adhered; light grey, not covered). [file Image_1.JPEG]
